# Status of Water Safety Plan Development and Implementation in Uganda

**DOI:** 10.3390/ijerph16214096

**Published:** 2019-10-24

**Authors:** Christopher Kanyesigye, Sara J. Marks, Juliet Nakanjako, Frank Kansiime, Giuliana Ferrero

**Affiliations:** 1 National Water and Sewerage Corporation, Plot 3 Nakasero, Kampala P.O. Box 7053, Uganda; Juliet.Nakanjako@nwsc.co.ug; 2 Eawag, Swiss Federal Institute of Aquatic Science and Technology, Überlandstrasse 133, 8600 Dübendorf, Switzerland; sara.marks@eawag.ch; 3 Makerere University, Department of Environmental Management, Kampala P.O. Box 7062, Uganda; fkansiime@caes.mak.ac.ug; 4 IHE Delft Institute for Water Education, Westvest 7, 2611 AX Delft, The Netherlands

**Keywords:** water safety plan, drinking water, risk assessment, audit, Uganda

## Abstract

Uganda was among the first countries in Africa that pioneered Water Safety Plan (WSP) development and implementation, with the first WSP dating back to 2002. The objective of this study was to assess WSP status in Uganda, focusing on the experience of the National Water and Sewerage Corporation (NWSC), in order to understand the factors that influenced it and strategies for scaling-up. This study consisted of a review of documentation for 20 WSPs, 42 interviews, a focus group discussion and four field visits. Results show that the development of the 20 WSPs over the last 15 years was largely incomplete and diverse. Most of the WSPs focused on system assessment and improvement, but failed to include WSP monitoring, verification and management. The monitoring of control measures was implemented in nine of the 20 systems, while verification took place in the form of internal (5/20) and external (2/20) auditing. The main barriers identified to WSP implementation were inadequate training, team composition and deployment, mistaken perception and inability to evaluate WSP effectiveness. Conversely, the main enabling factors were management commitment, public health responsibility, good customer relations, financial availability and reliable laboratories. These findings suggest a need for more institutionalization of WSPs with improved coordination across stakeholder groups.

## 1. Introduction

### 1.1. Water Safety Plans as a Key Tool for Sustained Safe Drinking Water Access

During the Millennium Development Goals (MDGs) period (2000–2015), considerable progress was made, particularly for Goal number 7, target c, which stated: “to halve the proportion of the universal population without sustainable access to clean and safe drinking water and basic sanitation by 2015”. By 2015, it was estimated that globally, the use of improved drinking water sources rose from 76 per cent to 91 per cent [1]. While most countries in developed regions achieved universal access, coverage varied widely in developing regions. In a bid to address the remaining gaps in drinking water service provision during the post MDG period (2015–2030), Sustainable Development Goals (SDG) Target 6.1 was set up that specifically aims at achieving universal and equitable access to safe and affordable drinking water for all by 2030. The achievement of this target is measured using indicator 6.1.1, which emphasizes the proportion of the population using safely managed drinking water services. Safely managed water is defined as an improved source located on premises, available when needed and free from faecal and priority chemical contamination [2]. The greatest share of the global population without sustainable access to safely managed drinking water is in the least developed countries. This particularly applies to sub-Saharan Africa where the estimated coverage in 2015, at the start of the SDG monitoring period, was below 25% [2].

The challenge of extending and maintaining access to safely managed drinking water services can be addressed through Water Safety Plan (WSP) development and implementation. Water safety planning emphasizes a preventative approach as the most effective means of consistently ensuring safe drinking water [3,4,5]. WSPs consist of a comprehensive risk assessment and risk management approach in all steps of a water supply, from catchment to consumers [6]. This process entails understanding and describing the complete water supply system, identifying where and how problems could arise, putting barriers and management systems in place to stop the problems before they happen, and making sure all parts of the system continue to function properly [6,7]. The objectives of WSPs are to prevent the contamination of raw water sources, treat water to remove contamination and prevent re-contamination during storage, distribution and handling [8]. 

### 1.2. WSP Development and Implementation in Africa

WSPs have been embraced globally and a few research studies indicated positive gains from their implementation [9,10,11]. On the African continent, however, the pace of WSP uptake has been slow and only a handful of research studies document this. As of 2017, 14 countries in Africa reported to have implemented WSPs [12], with Uganda being one of the pioneers of WSP development and implementation on the continent [13,14]. WSP development in Uganda started in 2002 and by 2009, implementation had been rolled out in 20 urban centers [9]. Local governments in some countries embraced WSP implementation. For example, in the Democratic Republic of Congo, the Healthy Villages and Schools (VEA) national program, was launched by the government as the main initiative to provide safe drinking water to rural and peri-urban populations. The program applied a WSP framework to ensure the delivery of safe and sustainable water services in schools and villages. In view of the poor drinking water quality observed at the household level, the promotion of household water treatment and safe storage practices was recommended [15,16]. In Kenya, the government, through the Ministry of Health issued a policy brief on water safety surveillance and recommended the training of all water service providers on WSP implementation as a means of averting frequent waterborne disease outbreaks in the country [17]. WSP development in Kenya started in 2009 with the training of selected managerial staff from Nairobi City Water and Sewerage Company and Mombasa Water Supply and Sewerage Company Limited. WSP development was later carried out in Kisumu Water Sewerage Company. The trainings were facilitated with support from the International Water Association [18]. In South Africa, an Emergency Response Plan (ERP) was designed by the Water Research Commission to ensure Water Safety and Security in South Africa. The ERP was developed through a field study of community water systems in the three provinces of Eastern Cape, KwaZulu-Natal and Northern Cape, whereby water supply emergencies were identified [19].

Some African countries particularly affected by climate change have established programs focused on minimizing its impact on water supply systems. In Ethiopia, climate-resilient WSP programs were adopted at the national level in response to water quality failures; by 2016, twelve WSPs had been implemented for urban and rural systems [12]. In the Comoros, a climate-informed water resources and watershed management program has been developed with funding from United Nations Development Programme (UNDP). The program focuses on developing planning guidance and procedures on source protection and water quality standards in view of climate change [20].

A few studies reported the adaptation of the WSP manual to suit local needs. In South Africa, Rand Water, one of the 15 water boards, spearheaded the development of customized WSPs by adding “procedure 11” to address support activities pertaining to customer focus, education, employee training and awareness, communication, research and technical support. The major support activities are carried out by government institutions and Non-Governmental Organizations (NGOs) responsible for catchment management [21]. In Senegal and Burkina Faso, WSP development and implementation in rural water supplies were launched in 2008 and 2011, respectively. Their implementation was carried out in a simplified manner, omitting some steps recommended in the WSP manual due to the absence of strong institutional support and the low technical expertise of the managers [22].

### 1.3. Sparse Evidence on the Benefits of WSPs in Africa and Beyond

The introduction and development of WSPs in Africa as described above has been ongoing for over a decade for some of the mentioned countries. It is, however, not possible to establish the full benefits of the WSP approach in any of these countries, since only two known studies have been carried out on WSP effectiveness in Africa, particularly in rural settings—one in South Africa and the other in the Democratic Republic of Congo (DRC). The study in South Africa made use of the Hazard Analysis and Critical Control Points (HACCP) approach for risk assessment from the point of delivery to households in small rural water systems in Limpopo province. The study showed that intermittent service delivery, access to unsafe water and poor household hygiene led to the consumption of contaminated water. It recommended the use of a HACCP approach to identify hazards and critical control points to reduce risks and attain improved safety at the household level [23]. The DRC study was part of a four-country research project carried out in India, DRC, Fiji, and Vanuatu to ascertain and share lessons learnt from the implementation of WSPs in community-managed water supply schemes. The study revealed that WSP implementation did not lead to improvement in microbiological water quality, but there was improved understanding of risk, water safety and security and in the development of improvement plans [24].

For the rest of the world, very few rigorous impact assessments of WSPs have been carried out. The earliest impact assessment of WSP implementation was carried out covering 16 water utilities in Iceland. This was after accumulated experience of WSP implementation by Icelandic water utilities following the enactment of WSPs in the drinking water legislation in 1995. Several benefits of the WSP approach were reported in the article published in 2012 including reduced risk from microbiological pollution, improved organizational culture and staff attitude [9]. In the Asia–Pacific Region, a study of the effectiveness of WSP implementation in 99 water supply systems across 12 countries was carried out in 2014–2016. This was the only study to present the results of WSP impact assessment based on the conceptual framework designed by the Center for Disease Control (CDC) and the results of audits. However, the study did not find statistically significant relationships between audit scores and the outcomes or impacts measured [11]. Finally, a study of the water quality, compliance and health outcomes of WSPs for utilities in France and Spain was also carried out for the period 2003 to 2015. Results indicated that the WSP approach may translate to diverse water quality, compliance, and health outcomes [10].

### 1.4. Purpose of this Study

The pace of WSP development and implementation in Africa has been slow and the lack of sustainable safe drinking water supplies remains a great challenge. There remains a need for empirical evidence regarding the factors behind the slow WSP uptake in the Africa region. The objective of this study was to evaluate the degree of WSP development and implementation in Uganda, focusing on the experience of the National Water and Sewerage Corporation (NWSC), to understand the factors that influenced it and strategies for scaling-up. The research questions that this study sought to answer are: (a) To what extent have all the WSP steps been covered and documented by each WSP team? (b) What are the factors that facilitated and hindered WSP development and implementation, i.e., enablers and barriers? (c) What are the roles and responsibilities of different actors in developing and implementing WSPs in Uganda? (d) What are the main intervention points needed to facilitate improved WSP performance and national scale-up? It is anticipated that small and large urban water supply practitioners and public health authorities in the developing world will find the results and recommendations of this paper useful for their respective mandates.

## 2. Materials and Methods

### 2.1. Study Area

From 2002 to 2004, the NWSC in collaboration with the Water, Engineering and Development Centre (WEDC) and Makerere University piloted WSPs in Kampala and Jinja with funding from the UK Department for International Development (DFID) [7,9,25]. By 2009, 20 towns under the jurisdiction of the NWSC had developed WSPs [9]. The NWSC also received support from the International Water Association (IWA) that facilitated the creation of an 18-month Water Operators Partnership (WOP) between the NWSC Jinja Area (branch) in Uganda, Kisumu Water and Sewerage Company (KIWASCO) in Kenya and the Mwanza Urban Water and Sanitation Authority (MWAUWASA) in Tanzania. Through the WOP, whose key objective was restoration of the Lake Victoria catchment, benchmarking visits between the utilities were performed. Document reviews and external audit also took place [26]. WSP implementation support was also received from the German Agency for International Cooperation (GIZ), which sponsored a consultancy for a review of WSP implementation in three NWSC Areas, namely, Entebbe, Masaka and Mbarara [26]. In Water Supply Systems (WSSs) that are not under the jurisdiction of the NWSC, WSPs have been developed by Umbrella Organizations (UOs) with support from the Austria Government [26]. The UOs are associations of Water Supply and Sewerage Boards supervised by the Department of Urban Water Supply and Sewerage Services, Ministry of Water and Environment (MWE) in Uganda.

Regarding the enabling environment, the Uganda National Bureau of Standards (UNBS) reviewed the Drinking (Potable) Water Specification (US 201:2008) by adapting the East African Drinking Water Standards in 2015, cited as US EAS 12:2014. According to the regulation, water production operators are required to develop, implement and maintain WSPs customized to the potential safety risks of the raw water supply area, the treatment plant, and the distribution network to the consumer points [27]. Currently, the MWE is in the process of developing a “National Framework for Management and Regulation of Drinking Water Quality in Uganda” that will include guidelines for water safety and security.

**Figure 1:**
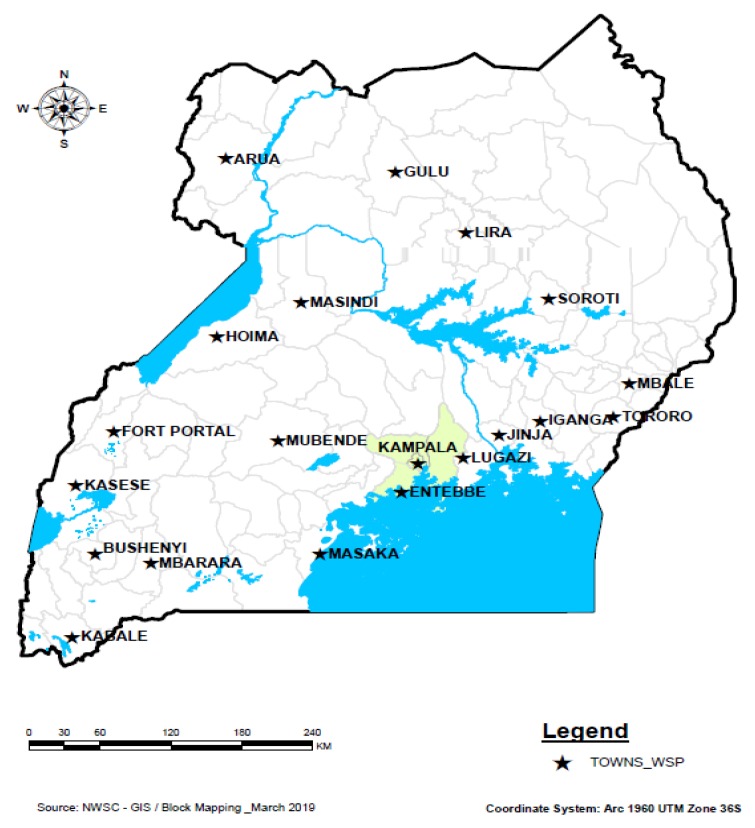
Location of the 20 Water Supply Systems (WSSs) managed by the National Water and Sewerage Corporation (NWSC) and implementing Water Safety Plans (WSPs).

WSP implementation has been taking place in 20 WSSs under the jurisdiction of the NWSC. All these WSSs were included in this study. The nature and size of business for each of the WSSs in terms of the type of raw water source, pipe network length, daily volume of water supplied, and number of consumer accounts are given in Table 1. The locations of the 20 WSSs across Uganda are shown in Figure 1.

### 2.2. Data Sources

This study made use of a mixed methods approach consisting of primary and secondary data sources. Primary data was collected via semi-structured interviews, direct observation during field visits and a Focus Group Discussion (FGD). The semi-structured interviews involved 11 top and middle managers and 24 staff from NWSC operational branches (areas) and 7 stakeholders from the National Environment Management Authority (NEMA), UNBS, WHO Country Office, Directorate of Water Development (DWD) of the MWE, Directorate of Environment of the MWE and Directorate of Water Resources Management (DWRM) of the MWE. The FGD involved 21 stakeholders from NWSC, MWE, UNBS and Makerere University. Secondary data sources were the WSP documentation of the 20 WSSs, internal and external WSP audit reports and water quality monitoring data for the period 2007 to 2017. We collated all available WSP documents and performed a systematic desk review. An internal informal audit was performed based on the WHO’s practical guidance manual [28]. Qualitative scores were used, whereby the maximum possible score for a WSS was 120 points arising from the total scores of the WSP development steps. From the WSP audit guide [28], each question may be scored 0 to 4 points. The number of questions for each step therefore determines its total score. The number of questions per development step is as follows: WSP team (4 questions), system description (4 questions), hazard identification and risk assessment (5 questions), implementation plan (3 questions), operational monitoring (2 questions), verification of WSP effectiveness (5 questions), prepare management procedures (3 questions), develop supporting programs (2 questions) and WSP review and revision (2 questions). The performance of a WSS was rated as “excellent” if its total score ranged from 115 to 120 points, “very good” for 103 to 114 points, “good” for 91 to 102 points, “average” for 79 to 90 points, “below average” for 61 to 78 points and “priority attention needed” for less than 61 points.

### 2.3. Data Preparation and Analysis

Data entry sheets for semi-structured interviews were designed prior to the interview process. Respondents were categorized as stakeholders, operators, top management and area teams. Themes such as funding schemes, levels of WSP development and implementation, success factors and barriers of WSP implementation and WSP scaling-up were defined in advance. After the interview process, the data was coded based on the variables under each research question and information sources. The interviewee responses were compared, contrasted and entered in the data sheets to calculate the percentage number of interviewees with similar and different opinions per study factor; the information was narratively described thereafter. The interview data were analyzed to obtain the main barriers and enablers of WSP development and implementation. FGD questions were prepared, vetted and agreed upon by the organizing team. The FGD process was recorded to back up the minutes of the proceedings and a report was prepared after consideration of the comments from the organizers. The results of the informal internal audit were analyzed using Microsoft excel to obtain the relative status of WSP development and implementation. 

### 2.4. Ethical Considerations

The research was conducted in accordance with the Declaration of Helsinki and the study protocol was approved by the Research Department of NWSC, Uganda, (Memo: BSS/R&D/17-11) as per government requirements. The research team sought approval from local leadership and informed consent from all respondents before conducting interviews and FGDs. Respondents’ information was treated with confidentiality, strictly for academic purposes.

## 3. Results

### 3.1. Findings of the Internal Audit

Results of the audit showed that overall no WSSs scored excellent, very good or good. Only three WSSs (Kampala, Jinja and Entebbe) attained an average score, four WSSs (Mbale, Soroti, Mbarara and Bushenyi) scored below average, and the rest needed priority attention (Table 2). 

A data file detailing the WSP audit scoring process for the 20 WSSs (Table S1) is provided as supplementary material.

The audit findings regarding WSP development and implementation steps were as follows:

***i. WSP team formation and stakeholder involvement:*** Approximately 50% of the WSSs (11/20) documented team formation. Apart from Kampala, Jinja and Entebbe, the lists were incomplete. For these three WSSs, the teams were largely multidisciplinary, having representation from both technical and support departments. Four WSSs, namely Kampala, Entebbe, Jinja and Gulu, documented stakeholder engagement, mainly in matters of catchment management, source protection and network maintenance. 

***ii. System description:*** Most WSSs (17/20) carried out and documented a system description of the water supply chain, although Masaka, Lira and Fort Portal did not include flow diagrams, and those for Hoima and Gulu were incomplete. Kabale, on the other hand, provided flow diagrams without the narrative description. 

***iii. Hazard identification and risk assessment:*** All 20 WSSs performed hazard identification and risk assessment, but of these, only four properly documented the hazardous events indicating the effects of the events on the water supply. Most of the WSSs did not indicate and describe the methodology for the rating of the risk components (likelihood and severity) and scoring and ranking of the risks. Regarding the hazardous events, some were not clearly described. In some instances, likelihood and severity ratings were exaggerated, leading to unrealistic risk scores. 

***iv. Determination of new control measures and improvement plans:*** All 20 WSSs determined and documented the new control measures needed to curtail risks and develop system improvement plans. The funding of the improvement plans was mainly linked to the annual budget of the respective WSS, although only eight WSSs explicitly indicated the source of funding. Regarding implementation of the improvement plans, most activities pertaining to water treatment and distribution were carried out since they fell within the annual budget and full mandate of the WSS (branch) concerned. However, those pertaining to catchment management and source protection were not all implemented due to inadequate stakeholder involvement and budgetary challenges. Examples include the monitoring of industrial effluent quality, construction and maintenance of urban drainage channels and enforcement of environmental pollution regulations. Overall, documentation of the improvement activities was not correspondingly updated except in Kampala, Entebbe and Jinja.

***v. Operational monitoring of control measures:*** Almost 50% of WSSs (9/20) documented and implemented a plan for the monitoring of control measures. The level of completeness of the operational monitoring plans, however, varied. To one extreme, one WSS only made a brief description of the activities without the parameters to be monitored and their critical limits, with no work plan attached. The most complete were the monitoring plans for Kampala, Entebbe and Jinja, where elaborate plans detailing what process steps to be monitored, when, how, by whom and the corrective actions to be made were documented. Regarding implementation, most WSSs did not fully follow the monitoring schedule described, except Kampala, Entebbe and Jinja. 

***vi. Verification:*** Approximately half of the WSSs (9/20) documented plans for WSP verification. Compliance monitoring was, however, not performed as scheduled, except in Kampala, where annual joint monitoring by the UNBS and MWE was performed. The audit found that all water quality standards and targets were met in two WSSs, namely Jinja and Entebbe, while most parameters were met in five of the WSSs. For the remaining WSSs, a number of the standards and targets were not met or data were not available. Internal and external audit schedules were made but not followed. Seven WSSs planned for WSP internal and external audits. Internal audits were, however, carried out in five locations, namely, Kampala, Jinja, Entebbe, Tororo and Mbale in 2008, while external audits were carried out in Kampala in 2008 and Jinja in 2013. The external audit conducted in Jinja was arranged under the WOP with Kisumu and Mwanza utilities with support from the IWA, while for Kampala, it was carried out by a PhD researcher from the University of Iceland [9]. Regarding consumer surveys, these were annually organized and carried out by the head office covering all the WSSs. 

***vii. Preparation of management procedures:*** Approximately half of the WSSs (9/20) prepared procedures for the management of the systems, including emergency situations. As noted from the WSP documents and during the field visits, some of the key Standard Operator Procedures (SOPs), e.g., for pump running, chemical mixing, doser control and water quality tests during routine treatment plant operations were printed and placed at visible points of the processes for ease of reference.

***viii. Development of supporting programs:*** Approximately half of the WSSs (9/20) developed and documented supporting programs as a means of sustaining WSP implementation. The main support programs implemented were staff training, regular maintenance and calibration of equipment, occupational health and safety programs and facilities for workers, cleaning schedules and various means of internal communication and customer engagement.

***ix. Periodic review and revision of the WSPs:*** Update of the WSP documents, apart from the initial documentation at inception, was performed, albeit irregularly. Ideally, a WSP review should be performed as frequently as the water supply system changes take place (e.g., weekly, monthly) and to respond to audit recommendations (e.g., every two years). For example, regarding system description and WSP teams: updating the system description and the risk assessment when new control measures are implemented and updating the WSP team when staff changes take place. In the case of incidents that substantially affect the performance of the water supply system, a revision of the WSP should be performed swiftly [6,26]. However, the audit revealed that the majority (12/20) of WSPs were updated fewer than three times in the past 15 years, indicating a generally low level of responsiveness to system changes. Additionally, only four WSPs, namely Kampala, Entebbe, Jinja and Gulu, had been recently updated, with most (16/20) not receiving an update in over four years (Table 3). Taken together, these results indicate that WSPs in Uganda had not been considered as a living document. 

### 3.2. Factors Influencing the WSP Status 

From the semi-structured interviews and field visits, the following factors were noted as either barriers or enablers of WSP development and implementation. 

#### 3.2.1. WSP Barriers

i. Non-Representative WSP Team Composition

Approximately 50% of the top management and WSP team members interviewed (6/11 and 10/20, respectively) stated that most staff believed that WSP implementation was a responsibility of staff from the technical departments, especially engineering and water quality management. Further inquiry showed that 65% of the WSSs (13/20) had complete WSP teams—the rest being incomplete with water quality and engineering staff as the only active members. Furthermore, it was established that inadequate mechanisms for the handover and training of transferred staff resulted in gaps of WSP teams—both in numbers and capacity.

ii. Inadequate Human Resources

Thirteen percent of top management and WSP team members (2/11 and 2/20, respectively) mentioned inadequate human resources to implement WSPs. Moreover, almost 50% of them said that staff transfers were not associated with a replacement with equivalent knowledge of WSPs. They expressed concern that such transfers hinder WSP continuity. On the other hand, when asked whether staff changes affected the implementation of WSPs, most top management members stated that it had no effect as long as the requirements of the human resource manual such as planned job rotation, transfer, enrichment and promotions were professionally followed.

iii. Inadequate Staff Training

Almost 50% of the WSP team members (9/20) expressed concern about the lack of knowledge among staff for WSP implementation, which could be attributed to inadequate training. This was confirmed by approximately one-third of them (6/20), who stated that not all operational staff were conversant with WSP implementation. Not surprisingly, only 5% of them stated that there was regular training on WSP development and documentation.

iv. Failure to Appreciate WSPs as a Worthwhile Approach

One-quarter of top management and WSP team members (3/11 and 5/20, respectively) stated that some managers did not appreciate WSPs as a worthwhile risk management approach—hence, they did not support and prioritize it. The failure to appreciate WSPs was because a few managers saw system water quality in compliance with national standards for most routinely monitored parameters—hence, they felt there was no need to fully implement WSPs.

v. Low Commitment by Managerial Staff to Invest in WSPs

Approximately 20% of the top management and WSP team members interviewed (4/11 and 2/20, respectively) responded that management was committed and willing to invest in WSPs. On the other hand, approximately 30% of them (4/11 and 5/20, respectively) reported poor commitment and willingness to invest and continually implement WSPs. Furthermore, several top management members expressed concern that some WSP teams did not continue with implementation after attaining training.

vi. Heavy Workload for WSP Documentation

Ten percent of WSP team members (2/20) stated that there was a lot of workload regarding WSP documentation on top of the periodic performance reports required. Furthermore, 20% of them (4/20) said that some activities such as revenue collection ventures were considered a priority, hence replacing the implementation of WSP assignments.

vii. Inability to Evaluate WSP Performance

Approximately 20% of top management and WSP team members (2/11 and 2/20, respectively) stated that it was difficult to evaluate the performance of WSPs. They said that WSP implementation benefits could not be quantified so as to help in soliciting management appreciation of WSPs.

#### 3.2.2. WSP Enablers

i. Public Health Responsibility

Over 50% of the top management and WSP team members (6/11 and 11/20, respectively) reported that the NWSC has a duty to provide safe drinking water to the public, which cannot be achieved without the implementation of WSPs. They stated that management provided support in terms of human resources and required finances in the case of water quality challenges in the operational areas.

ii. Good Customer Relations

Over 50% of the top management and WSP team members (8/11 and 10/20, respectively) stated good customer relations with staff, in line with NWSC’s motto: “the customer is the reason we exist”. They added that accordingly, staff try their best to build and sustain a good relationship with customers through providing services above expectations. A top management member stated that adhering to WSPs was the only way of providing potable water to the clients without losing their trust. He mentioned that in return, customers provided feedback on performance through the front desk offices and the central customer care center. A total of 50% of the top management members said that due to good customer relations, many more customer compliments were received than complaints.

iii. Enhanced Reporting Culture

Over 10% of the top management and WSP team members (2/11 and 2/20, respectively) said that all staff were continuously informed of the progress of events through social media platforms such as WhatsApp. They stated that the platforms facilitated the reporting of incidents and events in all branches openly and brainstorming on possible solutions.

iv. Enhanced Corporate Image

Almost 50% of the top management members (5/11) stated that the NWSC had a good public image due to the quality of service delivery and recognized WSPs as a means of maintaining the image.

v. Top Management Buy-in

Almost 50% of the top management members (4/11) said that there was WSP buy-in by the board of directors and top management. They stated that for that reason, as long as WSP requirements presented to top management are relevant and justified, they are approved for implementation. Such requirements include human resources, training needs and laboratory equipment.

vi. Planning and Financial Availability for WSPs

Approximately 30% of the top management and WSP team members (5/11 and 4/20, respectively) stated that funds were available for WSP implementation as long as responsible teams exercised timely budgeting. On the other hand, approximately 20% of them (2/11 and 4/20, respectively) said that a lack of funds hindered WSP implementation. Approximately 50% of the top management members, however, expressed concern over a lack of advance planning for the inclusion of WSP action plans into the annual budgets, making it difficult to facilitate their implementation. Apparently, 20% of the WSP team members (4/20) said that implementation of WSPs required an independent budget.

vii. Reliable Laboratories

Over 50% of the top management and WSP team members (7/11 and 10/20, respectively) stated that large WSSs had reliable laboratories, equipped for the detailed analysis of both microbial and physico-chemical water quality parameters. They added that detailed water quality analysis for small WSSs took place at regional laboratories to back up their limited process control and monitoring.

### 3.3. Strategies for Improving the Institutionalization and Scaling-Up of WSPs

Despite being included in the Ugandan regulation since 2008, water safety planning is a voluntary (not mandatory) undertaking under the law [27]. The need for making WSP implementation mandatory and strengthening enforcement was discussed by key actors such as the NWSC, MWE, Directorate of Water Development of the MWE, UNBS and Makerere University. According to the current legislation, the MWE does not have the legal mandate to enforce WSPs. Therefore, a new law should be approved to expedite WSP implementation. Awareness should be raised at higher levels within the Ministries in order to lobby for such a change. Another initiative for boosting WSP uptake may be the inclusion of WSPs in performance contracts with water utilities.

While discussing the costs involved in WSPs, there was consensus that the budget approved each year by the MWE should be derived from and informed by site-specific WSPs. The key actors recognized that the Regulation Division of the MWE should be the authority leading WSP external auditing, while NWSC and other water authorities should be in charge of internal auditing. The training and certification of WSP auditors was not discussed.

All participants expressed the need for a systematic documentation of experiences, demonstrating the benefits of WSPs and narrating success stories and challenges encountered during implementation. This will be instrumental for initiating the relevant moves at the policy level (i.e., institutional arrangements, legal requirements and enforcement mechanisms). At the implementer level, awareness is still missing: WSPs should be seen as a part of what is already performed during day-to-day operations; improving documentation will possibly help in conveying this message. Management commitment plays a crucial role in improving water safety.

Regarding the scaling-up process, different types of WSPs should be adopted depending on the complexity of the system and this should go hand in hand with templates that implementers could use (missing in the case of point sources). Peer-to-peer learning should be encouraged between utilities that are more proficient with WSP implementation and others that are starting up with WSP implementation or facing challenges.

Another key point explored was the need for enhanced coordination amongst the national stakeholders that are working on WSPs, linking the MWE and water utilities. Knowledge sharing should take place between different governmental organizations. Moreover, information about WSPs is only circulating among implementers but it should be reaching those in the Parliament that decide on budgets.

## 4. Discussion

### 4.1. Novelty and Contribution of the Study

WSP development and implementation in Africa has been ongoing for over a decade [12] and yet detailed information regarding successes and failures and the reasons behind them is not easily available. This study presents the first analysis of all 20 WSPs implemented by the NWSC in Uganda, consisting of piped schemes in cities and towns of varying sizes. The opportunity to deliver sustained safe water supply could be achieved with the adoption of WSPs in sub-Saharan Africa, where most water utilities, similar to NWSC, conduct microbial water quality monitoring but do not achieve regulatory standard targets [29]. Similar to the Asia–Pacific study, we employed primary and secondary data sources to systematically assess the status of the 20 WSPs and identify the main enablers and barriers that explain these audit scores. Even though audit scores may not be directly correlated to WSP outcomes and impacts [11], audit is a summative evaluation step that enables understanding knowledge application and synthesis [30]. This study serves as a benchmark for the assessment of WSP development and implementation in Africa, especially for water schemes serving small and large urban centers. This study also contributes to a growing evidence base regarding the status of WSPs globally, following their initial development and implementation.

### 4.2. Key Insights

This study revealed, first and foremost, that a wide range of actors across the water sector were involved in WSP development and implementation in Uganda. The actors ranged from research/university institutions (the WEDC and Makerere University at the initial formative stage) and international collaborators (the IWA and GIZ facilitating implementation) to government ministry and standards body (the MWE and UNBS for regulation) [7,9,25]. The international and national collaborators similarly facilitated WSP capacity building in Senegal and Burkina Faso [22], the Comoros [20], the Democratic Republic of Congo [15,16] and Kenya [17,18]. Similarly, a research project was carried out in four countries, including the DRC, to ascertain and share lessons learnt from WSP implementation with the support of UNICEF [31]. This suggests that the involvement of relevant external collaborators could be an important factor in sustained WSP implementation and scaling up WSP development in Africa.

Secondly, WSP documentation was largely incomplete as indicated by overall low scores from the internal audit. Just one-third of the 20 WSSs had a complete system description and all steps documented. The fact that a properly documented system description is the basis for an exhaustive hazard analysis, risk assessment and management, short of which a WSP is ineffective [32], could explain the failure to consistently meet the standards for drinking water quality for some WSSs in Uganda. The WSP global status report [33] remarked that maximum WSP benefits are realized when balanced attention is given to the entire WSP process. This covers the “front end” (team formation, system description, risk assessment and improvement plan) and the “back end” (process monitoring, verification, management and review) of the WSP process.

Furthermore, proper documentation of the hazardous events and risk rating is critical in determination of the appropriate control measures at all stages of the supply chain [6]. The control measures once established have to be monitored by following a documented monitoring schedule involving onsite observations and/or laboratory analysis. A properly implemented monitoring plan helps to save on laboratory water quality analysis, as only confirmatory tests may be required [7,14]. Regarding verification, the gap in compliance monitoring and external audit could be bridged through increased partnership with regulatory bodies mandated to carry out surveillance, namely the Ministry of Health, MWE-DWRM and UNBS. The irregular WSP auditing observed is not aligned with the WHO’s statement that auditing is critical for sustainable WSPs as a check for completeness, adequacy and effectiveness, while supporting iterative adjustments and continuous improvement [28]. Another critical finding of the audit was irregular review and revision of the WSP documentation irrespective of long periods of system changes. Ideally, a review and update of WSP documents needs to be performed regularly to respond to changes in the water supply system [33].

Several factors were noted as barriers to WSP development and implementation. The basic principles of WSPs require that a team with experience and expertise on the entire water supply chain is formed to spearhead WSP development and implementation [6]. In this study, it was apparent that incomplete and non-representative WSP teams resulted in inadequate WSP documentation and implementation in most of the WSSs. Coupled with proper team formation is the need for WSP training which was not adequately performed as expressed by approximately one-third of the interviewed operations team members. Studies have shown that capacity building activities such as training greatly improve WSP implementation and scaling-up [4]. Studies have also shown that, within the utility, initial training plus follow-up is crucial for both continuing and new personnel [30]. Moreover, methods to monitor WSP capacity development should be developed and strengthened [30]. The lack of WSP awareness and guidance hinders WSP implementation [8,34,35]. For instance, some managers did not appreciate WSPs as a worthwhile risk management approach, stating that the quality of water supplied already complies with National standards for most routinely monitored parameters. This, however, indicates a lack of knowledge that WSPs are a preventive risk management program meant to sustain the good quality water supply [6]. Moreover, the audit results disqualified the belief revealing that water quality results for 13 out the 20 WSSs did not consistently comply with the national drinking water standards. Another key barrier to WSP implementation was the inability to evaluate WSP performance. This is due to failure to implement the WSP verification steps that involve compliance monitoring and auditing. There was therefore failure to quantify some of the WSP benefits that could enhance its appreciation. A number of authors have also found that there was difficulty in evaluating WSP implementation despite the existence of an evaluation framework [36] and its application, e.g., in Iceland, France, Spain and in the Asia–Pacific Region [9,10,11].

Nonetheless, enablers to successful WSP development and implementation were also identified, including public health responsibility that entails the provision of potable water, which may not be consistently achieved without WSPs. This was similarly reported by Rand Water in South Africa, which stated that commitment to water quality among staff was one of the factors enabling successful WSP implementation [37]. Relatedly, good customer relations enhanced WSP implementation [38]. Various reporting fora including social media platforms such as WhatsApp and Twitter, sometimes involving both staff and customers, were noted as WSP enablers. Through the convenient platforms, the communication of events, a culture of positive criticism as well as embracing new ideas promotes sustainable WSPs [35,39]. Customer engagement also leads to enhanced corporate image, particularly making use of the central call center, where compliments and complaints are received and relayed and feedback is provided. Similar findings were recorded among water utilities in South-East Asia and Europe [8]. The availability of reliable laboratories for water quality monitoring was also noted to enable WSP implementation as previously remarked in the external audit of Kampala water supply [9].

### 4.3. Limitations

There are several aspects of the design of this study that limit its generalizability beyond the schemes examined. First, this study focused on piped WSSs in 20 medium to large urban centers under the jurisdiction of the NWSC that are predominantly characterized by conventional surface water treatment, with the exception of only two WSSs relying on groundwater sources. Urban water supply systems in Uganda currently include a large number of very small piped systems with groundwater sources. Most peri-urban and rural water supplies on the other hand are non-networked community-managed water points (e.g., boreholes equipped with hand pumps). The findings of this study may therefore not fully apply to the entire Ugandan water supply infrastructure system. Furthermore, the audit part of this study was dependent on secondary data that may not have provided a complete picture of the reality of WSS management and operation. Such pre-existing data sources are prone to the positivist fallacy that the absence of evidence is evidence of its absence, which in fact may be untrue. For example, in this study, we examined water quality data only regarding compliance for the period of 2007 to 2017. In addition, this study is limited in scope to the WSPs’ implementation status and their associated enablers and barriers. We did not analyze whether water quality outcomes were better for WSSs with better WSP implementation. Finally, the audit scores generated in this study relied on the method promoted by the WHO practical guide to auditing WSPs that is widely used in the sector. However, it is possible that this auditing approach over or under values certain measures in terms of their actual influence on WSS functionality and service outcomes.

### 4.4. Key Message for Practitioners and Policy Makers in the Water Sector in the Developing World

To overcome the implementation barriers and harness the enabling factors revealed in this study, WSPs must be institutionalized as part of the day-to-day operations of water utilities. Knowledge sharing and peer-to-peer learning should be encouraged to boost capacity among utilities in developing countries. The active involvement of relevant stakeholders in WSP development and implementation needs to be ensured with the utility taking the lead in implementation and regulators spearheading surveillance and verification.

For WSP sustainability, there’s a need for the identification of qualified and experienced multidisciplinary teams to champion WSP development and implementation. Furthermore, there’s a need for the regular training of the teams, managers and other staff to facilitate factual decision making, maintain capacity and promote WSP scale-up. There’s also a need to invest more in laboratory infrastructure to facilitate process and water quality monitoring to achieve quality assurance. Equally critical is the need to carry out a periodic WSP performance evaluation in order to quantify WSP benefits that could enhance its effectiveness, sustainability and scale-up.

## 5. Conclusions

This study revealed that despite WSPs being introduced in Uganda over 15 years ago, and gradually developed and implemented thereafter, the level of their implementation remained low. Only three WSSs attained an average audit score, four WSSs scored below average, and 13 WSSs needed priority attention. Most of the 20 WSSs carried out system assessments and put in place improvement plans, but just over one-third had documented and carried out process monitoring, verification in terms of auditing and compliance monitoring, and periodic review. These results reflect a gap in management teams’ understanding and application of the basic WSP principles as a preventive risk management approach for piped water supplies serving small towns in Uganda. This study identified several barriers to the thorough implementation of WSPs in this context, including the inadequate training of management and staff, low awareness of the benefits of WSPs and incomplete team composition and deployment. Interviews also revealed competing management priorities and an inability to evaluate WSP effectiveness as key barriers to WSP implementation. On the other hand, several enabling factors were identified as aiding WSP implementation, including top management commitment, public health responsibility, good customer relations and public image, enhanced reporting culture, availability of adequate funds and reliable laboratories. Efforts to specifically improve staff training, awareness and promotion of management commitment, customer relations procedures, reporting and budgetary provision could result in improved and sustained WSP implementation. Further research should focus on evaluating the impacts of WSPs in Sub-Saharan Africa, especially under varying degrees of implementation. Future investigations should also examine the applicability of standard indicators for measuring the outcomes of WSP in this context. This will provide the evidence base needed in support of the appropriate enforcement, investment and upscaling of WSPs not only in Uganda, but in the developing world at large.

## Figures and Tables

**Table 1:** Characteristics of the 20 WSSs.

WSS	Source Type	Pipe Network Length (Km)	Water Supplied, m^3^/d (June 2017)	Number of Water Accounts (June 2017)
Arua	River	220	3314	7085
Bushenyi	Wetland	561	3317	9444
Entebbe	Lake	419	17,484	32,134
Fort Portal	River	220	3060	8637
Gulu	Dam	190	3025	6832
Hoima	Ground	152	1704	5012
Iganga	Lake	160	681	8436
Jinja	Lake	647	18,939	24,792
Kabale	Lake	214	1959	6125
Kampala	Lake	3001	207,730	255,203
Kasese	River	217	3099	8073
Lira	Lake	240	5681	10,750
Lugazi	Ground	84	1108	2501
Masaka	Wetland	360	6850	14,958
Masindi	Lake	187	2007	4915
Mbale	River	583	6282	15,619
Mbarara	River	485	12,963	18,555
Mubende	Dam	129	1661	4585
Soroti	Wetland	228	2917	7162
Tororo	River	288	2793	9274

**Table 2:** Summary informal WSP audit results for the 20 WSSs.

WSP Element	WSP Team	System Description	Hazard Identification and Risk Assessment	Improvement Plan	Operational Monitoring	Verification of WSP Effectiveness	Prepare Management Procedures	Develop Supporting Programs	WSP Review and Revision	Total Score	Rating
Maximum score	16	16	20	12	8	20	12	8	8	120	
Jinja	8	16	17	9	7	13	7	7	5	89	Average
Kampala	8	14	17	10	7	12	7	7	5	87	Average
Entebbe	8	11	17	9	7	12	7	5	5	81	Average
Mbale	5	15	15	9	6	9	6	6	2	73	Below average
Mbarara	0	14	13	10	4	9	7	7	3	67	Below average
Soroti	5	13	14	9	6	5	7	6	2	67	Below average
Bushenyi	0	16	14	9	4	9	7	3	1	63	Below average
Arua	2	10	13	9	4	4	7	4	1	54	Priority attention needed
Tororo	0	10	12	9	4	4	8	4	0	51	Priority attention needed
Hoima	5	8	13	10	0	0	0	0	0	36	Priority attention needed
Gulu	0	12	13	8	0	0	0	0	0	33	Priority attention needed
Kasese	0	11	12	8	0	0	0	0	0	31	Priority attention needed
Lira	5	4	12	9	0	0	0	0	0	30	Priority attention needed
Masindi	3	3	12	9	0	2	0	0	0	29	Priority attention needed
Masaka	0	7	13	8	0	0	0	0	0	28	Priority attention needed
Fort Portal	0	6	12	8	0	0	0	0	0	26	Priority attention needed
Kabale	0	3	11	9	0	3	0	0	0	26	Priority attention needed
Lugazi	5	0	12	8	0	0	0	0	0	25	Priority attention needed
Iganga	4	0	11	9	0	0	0	0	0	24	Priority attention needed
Mubende	0	0	13	8	0	0	0	0	0	21	Priority attention needed

**Table 3:** Number and years of WSP documentation updates.

WSS	Year of WSP Development	Year of WSP Update	
		1st	2nd
Jinja	2002	2013	2017
Kampala	2002	2012	2017
Entebbe	2005	2009	2017
Mbale	2004	2010	2012
Mbarara	2006	2009	2012
Soroti	2006	2009	2012
Bushenyi	2009	2012	
Arua	2009	2012	
Tororo	2004	2009	2012
Hoima	2009	2013	
Gulu	2006	2012	2017
Kasese	2009		
Lira	2006	2013	
Masindi	2009	2012	
Masaka	2005	2012	
Fort Portal	2006	2012	
Kabale	2007	2009	
Lugazi	2009		
Iganga	2009		
Mubende	2009	2012	

## Supplementary Materials

The following file is available online at https://www.mdpi.com/1660-4601/16/21/4096/s1, Table S1: Water Safety Plan audit scoring process for the 20 water supply systems.
